# Changes of Brain Glucose Metabolism in the Pretreatment Patients with Non-Small Cell Lung Cancer: A Retrospective PET/CT Study

**DOI:** 10.1371/journal.pone.0161325

**Published:** 2016-08-16

**Authors:** Weishan Zhang, Ning Ning, Xianjun Li, Gang Niu, Lijun Bai, Youmin Guo, Jian Yang

**Affiliations:** 1 Radiology Department of the First Affiliated Hospital, Xi'an Jiaotong University, Xi'an, Shaanxi, People's Republic of China; 2 Nuclear Medicine Department of the Second Affiliated Hospital, Xi'an Jiaotong University, Xi'an, Shaanxi, People's Republic of China; 3 Department of Biomedical Engineering, the Key Laboratory of Biomedical Information Engineering of the Ministry of Education, School of Life Science and Technology, Xi'an Jiaotong University, Xi'an, Shaanxi, People's Republic of China; Catalan Institute of Oncology, SPAIN

## Abstract

**Objective:**

The tumor-to-brain communication has been emphasized by recent converging evidences. This study aimed to compare the difference of brain glucose metabolism between patients with non-small cell lung cancer (NSCLC) and control subjects.

**Methods:**

NSCLC patients prior to oncotherapy and control subjects without malignancy confirmed by 6 months follow-up were collected and underwent the resting state 18F-fluoro-D-glucose (FDG) PET/CT. Normalized FDG metabolism was calculated by a signal intensity ratio of each brain region to whole brain. Brain glucose metabolism was compared between NSCLC patients and control group using two samples t-test and multivariate test by statistical parametric maps (SPM) software.

**Results:**

Compared with the control subjects (n = 76), both brain glucose hyper- and hypometabolism regions with significant statistical differences (*P*<0.01) were found in the NSCLC patients (n = 83). The hypermetabolism regions (bilateral insula, putamen, pallidum, thalamus, hippocampus and amygdala, the right side of cerebellum, orbital part of right inferior frontal gyrus and vermis) were component parts of visceral to brain signal transduction pathways, and the hypometabolism regions (the left superior parietal lobule, bilateral inferior parietal lobule and left fusiform gyrus) lied in dorsal attention network and visuospatial function areas.

**Conclusions:**

The changes of brain glucose metabolism exist in NSCLC patients prior to oncotherapy, which might be attributed to lung-cancer related visceral sympathetic activation and decrease of dorsal attention network function.

## Introduction

The neurobiological view of cancer aetiopathogenesis suggests that cancer information is conveyed by neural and humoral pathways to the special brain structures, and that brain might consequently modulate the neuroendocrine-immune system for response to the growth of tumors [1–5]. The previous studies on brain abnormal activation patterns associated with lung cancer have described the potential mechanism underpinning the cancer neuromodulation [2, 5, 6]. Since the afferent signal from tumors located in peripheral tissues is integrated to a number of brain regions [1, 3], thus the regional abnormalities in the brain mapping of lung cancer patients may reveal the tumor-related neuromodulation mechanism.

Metabolic imaging techniques such as magnetic resonance spectroscopy (MRS) and positron emission tomography/computed tomography (PET/CT) have documented significant changes in the metabolic and functional status in the resting-state brain of patients with lung cancer. In a MRS study, it was shown that the concentrations of glutamate, creatine and phosphocreatine in the parietal and occipital cortex were lower in patients with lung cancer prior to treatment, which indicated decreased neuronal metabolism [2]. The fluorodeoxyglucose (FDG), an analog of glucose labeled with positron emitting radioisotope (^18^F), indicates tissue metabolic activity by virtue of the regional glucose uptake. The ^18^F-FDG PET/CT studies showed that there were a reduced prefrontal activation [7, 8], and higher glucose metabolic rate in the right part of the cerebellum in lung cancer patients [5]. However, treatments of tumor, such as chemotherapy and surgery, could also influence brain activities [9–11]. Studies on patients without any chemotherapy, radiotherapy, or surgery might show the original activation of brain structures in response to the growth of cancer.

The objective of our study was to clarify the regional brain glucose metabolic abnormalities in patients with non-small cell lung cancer (NSCLC) using ^18^F-FDG PET/CT. All patients included in this study were evaluated according to neoplasm staging and pathological type before further treatments such as surgery, radiotherapy, or chemotherapy.

## Methods

### Study population

This single-center retrospective study was approved by the Ethics Committee of the First Affiliated Hospital of Xi’an Jiaotong University, and the written consent was obtained prior to scanning. This study was conducted in accordance with the Declaration of Helsinki.

All information of patients were anonymized and de-identified prior to analysis. From May 2012 to October 2014, 239 patients with NSCLC were consecutively enrolled. All patients were of Chinese Han ethnicity and were histopathologically diagnosed by pneumocentesis, surgery, or thoracoscope within a week after the PET/CT examination. Subjects with fasting plasma glucose levels higher than 6.1 mmol/L, poor image quality which was characterized by graininess of the liver [12] or PET/CT examination more than one week were excluded. Other exclusion criteria included brain tumors (primary brain tumor or metastasis), prior surgery, radiotherapy or chemotherapy, stroke, head trauma, hypo-or hyperthyroidism, diabetes, renal failure, chronic hepatopathy, chronic heart disease, autoimmune diseases, history of alcohol dependence, or acute and chronic infectious diseases. In this study, the subjects were excluded if it had a history of mental illness(insomnia, depression, mania, schizophrenia and other mental diseases) or a medical history of drug dependence (such as sedatives, sleeping pills or sedatives). The control group comprised those who underwent whole-body PET/CT scan for the first time in order to screening tumor, and showed no evidence of malignancy in the examination. All control subjects were further confirmed by follow-up visit for 6 months after PET/CT examination. The same exclusion criteria as the lung cancer group were applicable to the control group.

### PET/CT acquisition

^18^F-FDG PET/CT was performed with a resting state. PET data of the brain were acquired using a clinical PET/CT scanner (PHILIPS GEMINI TF 64-PET/CT). Plasma glucose levels were verified prior to FDG administration. Participants underwent fasting for at least 8 hours before being injected with 3.7 MBq ^18^F-FDG per kilogram of body weight (220~440 MBq) and then rested for 60 min in a quiet room with dim light and eyes opened. They were instructed to refrain from reading, listening to music, and talking during the uptake period. The brain scan consisted of a simultaneous CT scan and a 7-min PET study, and performed at 60 min after ^18^F-FDG injection. A separate whole-body PET/CT scan was used for diagnosis before brain scan. Fully three-dimensional mode PET data from the brain scan (90 slices) were reconstructed using a line-of-response row-action maximum likelihood algorithm (LOR-RAMLA) as 128×128 pixel images with a pixel size of 2 mm×2 mm and a slice thickness of 4 mm. Brain CT scan parameters: voltage 120 kV, current 240 mA, thickness 5 mm, and the CT data were used for PET attenuation correction.

### Data analysis

Statistical parametric mapping 8 (SPM8) (Wellcome Department of Cognitive Neurology, Institute of Neurology, University College London) implanted in MATLAB 7 (The MathWorks, Natick, MA) was used for image processing. PET images were interpolated to a 2 mm×2 mm×2 mm voxel size (trilinear interpolation), spatially normalized to the standard PET template embedded in SPM8, adjusted to the Talairach stereotactic brain atlas, and further smoothed with a Gaussian kernel of 5 mm in full width at half maximum. Global normalization and proportional scaling with 0.8 threshold masking were used. Coregistration of PET and MRI T1WI was the first step to combine the functional information from PET with anatomical information in MRI. Through the SPM routine, the mean error of translation in x, y, and z direction was below 1 mm. The standard deviations of the translation errors were small throughout all the simulated PET data sets. The mean error of pitch, roll, and yaw estimates was smaller than 0.6 degree and often close to 0 degree [13]. All the images of individuals were spatially normalized to the template spaces. The transformation parameters were derived from the normalization of the T1WI MRI images to the Anatomical Automatic Labeling (AAL, Montreal Neurological Institute) template. The PET images were normalized according to the transformation parameters of the corresponding T1WI. The brain was segmented into 116 brain regions according to the AAL atlas. The mean signal intensity (SI) per pixel in every brain region and the whole brain was estimated, and then the Normalized FDG metabolism (SI_region_/SI_whole brain ratio_) in every region was obtained.

Statistical analysis was conducted using SPSS for Windows version 17.0 (SPSS, Chicago, IL, USA). Measurement data were reported as means ± standard deviations or medians with ranges, and categorized as frequencies and percentages. The two-sample *t-*test was used for group comparisons between the control subjects and patients with NSCLC. A multivariate analysis using a general linear model was performed to investigate an effect of clinical stages and pathological types (only for adenocarcinoma and squamous carcinoma groups) on the uptakes of 116 brain regions with age and gender as covariates. *P*<0.01 was considered to be statistically significant. The significant results of abnormal glucose metabolism regions were overlaid on MR image in order to vividly display.

## Results

**Fig 1:**
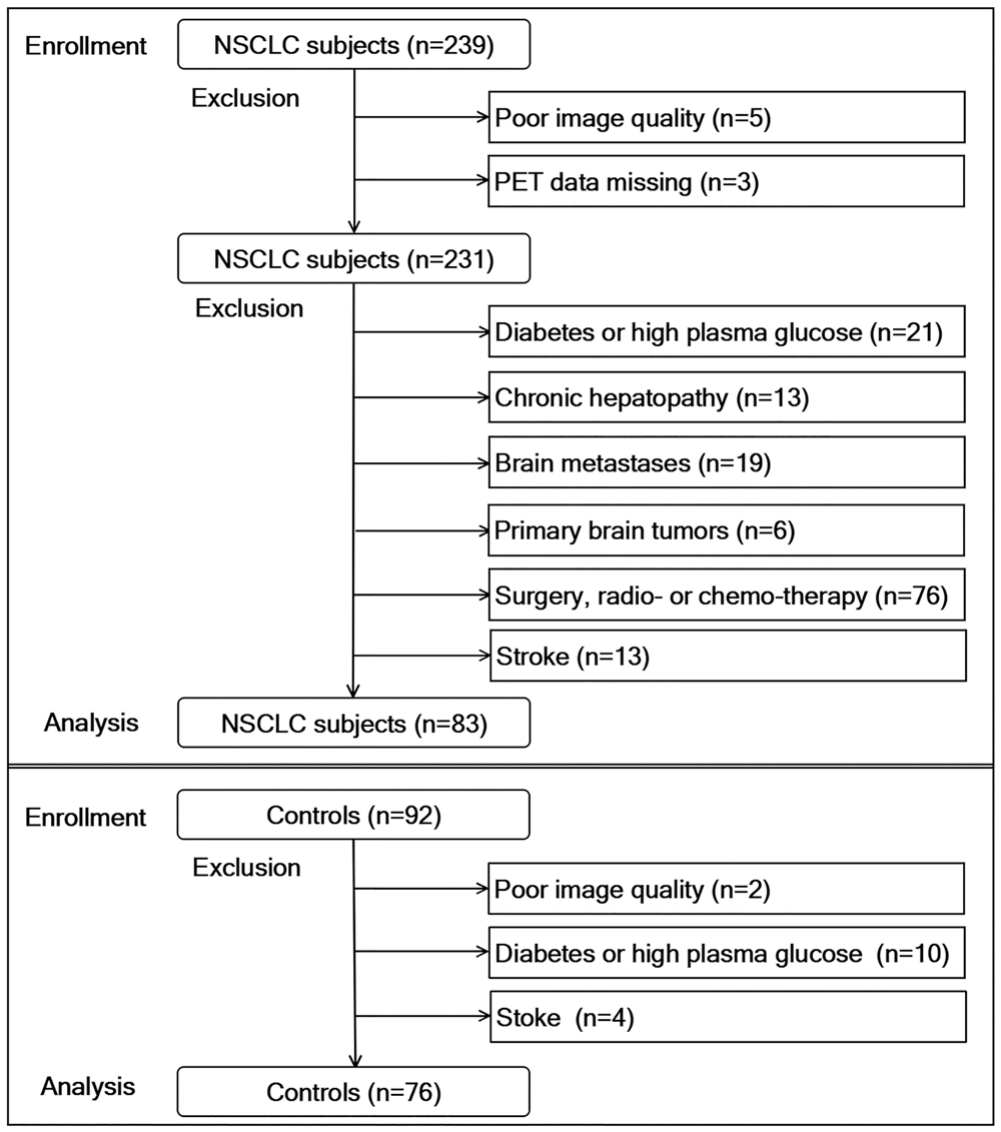
Flow chart of study population selection and the inclusion and exclusion criteria. Three PET/CT data of 239 NSCLC subjects were missing and the PET/CT image quality was poor for 5 subjects. Patients with diabetes or high plasma glucose (n = 21), chronic hepatapathy (chronic hepatitis B, n = 13), brain metastases (n = 19), primary brain tumors (gliomas,n = 2; meningiomas,n = 4), prior surgery, radiotherapy or chemotherapy (n = 76), or stroke (n = 13) were excluded. Controls with diabetes or high plasma glucose (n = 10), history of stroke (n = 4) or poor image quality (n = 2) were excluded. This left a total of 83 patients and 76 controls for analysis.

The study comprised 239 patients with lung cancer and 92 control subjects. Patients with diabetes or high plasma glucose (n = 21), chronic hepatitis B (n = 13), brain metastases (n = 19), primary brain tumors (gliomas,n = 2; meningiomas,n = 4), prior surgery, radiotherapy or chemotherapy (n = 76), history of stroke (n = 13), patients with missing PET data (n = 3) or poor PET/CT image quality (n = 5) were excluded in this study (Fig 1). The controls with diabetes or high plasma glucose (n = 10), history of stroke (n = 4) or poor image quality (n = 2) were excluded. Finally, 83 patients with NSCLC and 76 control subjects were enrolled in this study.

Distribution of selected characteristics between NSCLC patients and control subjects is summarized in Table 1. Baseline characteristics such as gender, age, plasma glucose level, race, and body mass index showed no significant differences between 2 groups. The patients were categorized based on the clinical stage of lung cancer (7th Edition of TNM Staging, UICC, 2009) and the pathological types and clinical stages in NSCLC patients were showed in Table 1.

**Table 1:** Distribution of characteristics among patients and controls.

Variables	Patients (n = 83)	Healthy Controls (n = 76)	*p*
	Mean or Count (%)±SD	Mean or Count (%)±SD	
Age	53.65±7.81	51.83±8.01	0.15*
BMI,kg/m^2^	25.88±3.61	27.31±2.89	0.12*
Plasma glucose,mmol/L	5.23±0.52	5.19±0.50	0.63*
Gender (Male)	59 (71.08%)	51 (67.11%)	0.59^#^
Smoker	52(62.65%)	42(55.26%)	0.35^#^
Pathology Type			
AC	51	-	-
SCC	29	-	-
BAC	3	-	-
Stage			
Stage I	16(19.3%)	-	-
Stage II	15(18.1%)	-	-
Stage III	28(33.7%)	-	-
Stage IV	24(28.9%)	-	-

**Abbreviations:** AC, adenocarcinoma; BAC, bronchioloalveolar carcinoma; BMI, body mass index; SCC, squamous cell carcinoma; SD, standard deviation.

* indicates independent-samples T test;

^#^ indicates Pearson Chi-Square Test.

**Fig 2:**
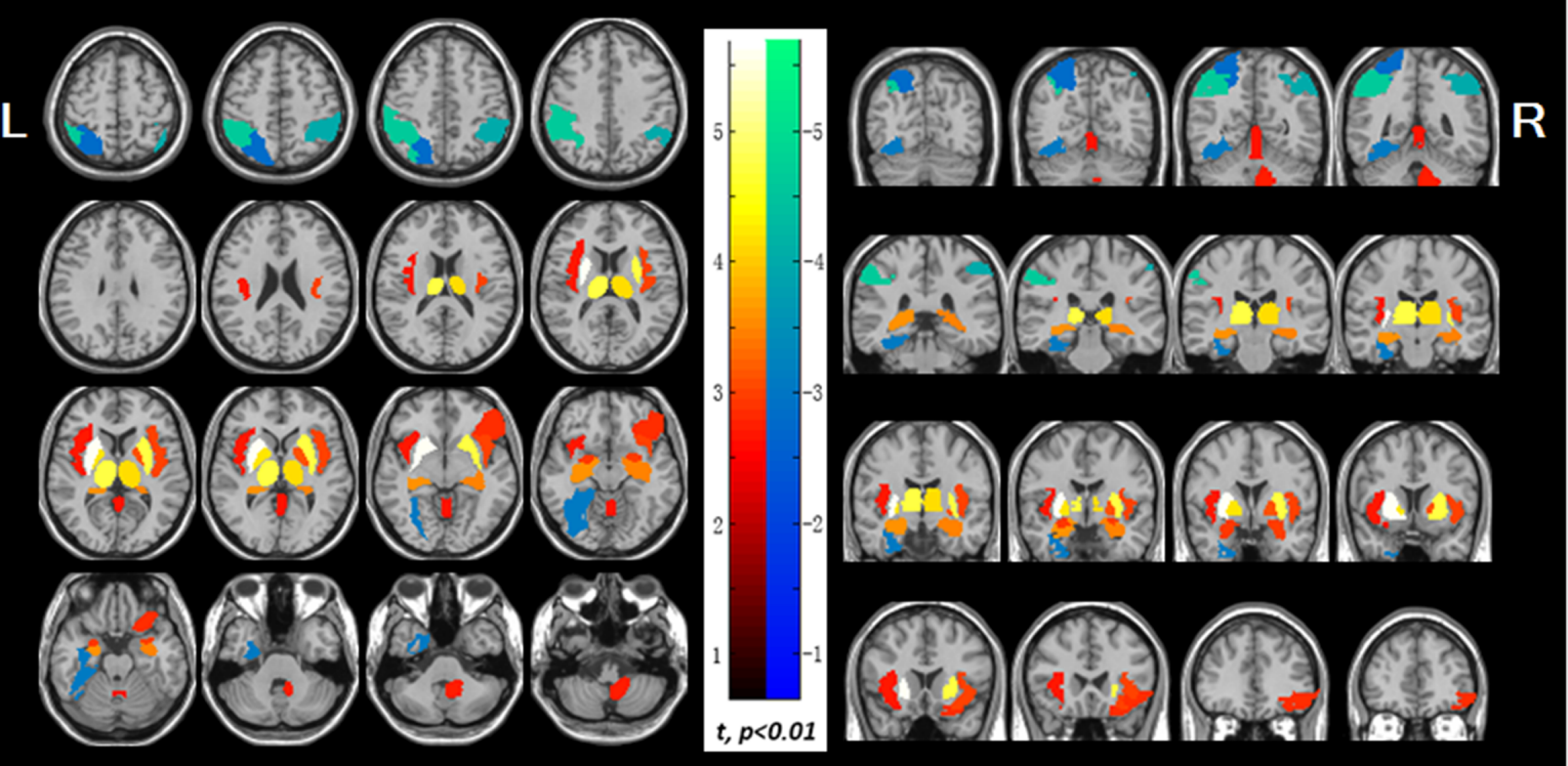
Abnormal glucose uptake in the non-small cell lung cancer patients. The increased regions display with red-to-white colour, which include both left and right sides of the insula, putamen, pallidum, thalamus, hippocampus and amygdala, the right side of cerebellum, orbital part of right inferior frontal gyrus and vermis, while the decreased regions display with blue-to-green colour, which include left superior parietal lobule, bilateral inferior parietal lobule, and left fusiform gyrus (p<0.01). PET findings were overlaid on magnetic resonance image secondary. Color bar indicates t-values; L: left; R: right.

The results showed that both decreased and increased brain glucose uptake were observed in patients with NSCLC (Table 2 and Fig 2). In Fig 2, the PET findings were overlaid on magnetic resonance image. The increased regions included both sides of the insula, putamen, pallidum, thalamus, hippocampus, and amygdala, the right side of cerebellum, orbital part of right inferior frontal gyrus, and vermis, which were the central components of the tumor-to-brain pathway and centers in the brain of viscerosensory paths [3, 14, 15]. The decreased regions were mainly located in the left superior parietal lobule, bilateral inferior parietal lobule, and left fusiform gyrus, which were the nodes of dorsal attention network (DAN) [16]. In the multivariate analysis, no significant differences of brain glucose uptake were found among the clinical stages or between adenocarcinoma and squamous carcinoma groups.

**Table 2:** Brain regions (AAL template) of abnormal glucose uptake in non-small cell lung cancer patients.

Type of uptake	Regions	R/L	*t*(df = 157)	Sig.(2-tailed)
**Increased uptake regions**	Insula	L	2.675	0.008
		R	3.118	0.002
	Putamen	L	6.461	<0.001
		R	5.400	<0.001
	Pallidum	L	4.645	<0.001
		R	3.248	0.001
	Thalamus	L	5.293	<0.001
		R	4.623	<0.001
	Hippocampus	L	3.919	<0.001
		R	3.750	<0.001
	Amygdala	L	3.002	0.003
		R	3.046	0.003
	Inferior frontal gyrus	R	2.887	0.004
	Cerebelum-10	R	2.846	0.005
	Vermis-4-5	-	2.666	0.008
**Decreased uptake regions**	Inferior parietal lobule	L	-5.285	<0.001
		R	-4.335	<0.001
	Superior parietal lobule	L	-2.830	0.005
	Fusiform gyrus	L	-3.184	0.002

**Abbreviations:** L, left; R, right; sig, significance;df, degrees of freedom. Increased and decreased glucose uptake in the non-small cell lung cancer patients. *t* indicates *t* values in the two-sample t-test between the control subjects and patients.

## Discussion

In this study, the abnormalities of brain glucose metabolism were observed in the NSCLC patients without oncotherapy. Compared with the control subjects, the abnormal brain regions mainly included the cerebellum, diencephalon, basal ganglia, limbic system, and particular cortical areas, which related to the visceral to brain signal transduction pathways and the DAN. The abnormal ^18^F-FDG mapping in the brain of NSCLC patients may reveal the primarily interaction between central neuromodulation and tumor-related peripheral reaction.

### Increased brain glucose uptake in NSCLC patients

The findings of this study further support the report from Golan and colleagues regarding hyperactivity in the right cerebellum and potential role of the vagus in tumor-to-brain communication [5]. According to the previous reports, the cerebellum plays an important role in immune regulation and responds to peripheral inflammation due to its proximity to the nucleus tractus solitarii (NTS), which is innervated by the vagus [17, 18]. The hyperactivity in the right cerebellum was considered to be associated with the tumor-related immune regulation. In the current study, the FDG hypermetabolism in the vermis was also observed, which is in proximity to periaqueductal gray matter (PAG). The PAG and NTS play crucial roles in the visceral influences to the brain and regulation of immune responses in the brain [13, 19]. This result could be the complement of Golan's study. On the other hand, treatments of tumor such as chemotherapy and surgery could also influence brain activities [9–11]. The control group in the Golan’s study enrolled patients with lymphoma and those patients had already received chemotherapy. Hence, the malignancy and treatments of tumor related changes in brain function [20] might be ignored in this previous study.

Since anxiety-related arousal might be less prominent in NSCLC patients who have already been scanned multiple times, all subjects consisted only of participants scanned for the first time in our study. The control subjects without malignant tumor as a suitable reference were in favour of detection of abnormal glucose metabolism in brain of NSCLC patients. Therefore, the extra abnormal metabolism regions of the cerebellum were observed.

Unlike Golan’s study, our data showed other brain regions of hyperactivity in the right inferior frontal gyrus, insula, basal ganglia, thalamus, and amygdala. These regions are involved in the primary sites of visceral cortex and subcortical nucleus [14, 15], and the main sites of the visceral to brain signal transduction pathways [21]. Furthermore, cancer information is conveyed centrally by cranial and spinal nerves within special brain structures, and brain might consequently modulate the neuroendocrine-immune system to regulate the growth of tumor in peripheral tissues [3]. These higher metabolism regions may reflect an attempt to reinstate homeostasis in functions such as respiration and immunity pertinent to lung malignancy and relate to the visceral sympathetic activation [5].

Besides, hippocampus and amygdala on behalf of the emotion-related regions showed the increased brain glucose uptakes in this study. Previous studies have reported that the patients with malignant tumor usually have complications such as emotional distress and psychiatric syndromes. Diagnosis of malignant tumor is a huge stress to patients and causes the fear, anxiety, anger and sadness [20, 22, 23]. A voxel-based meta-analysis of 105 functional magnetic resonance imaging (fMRI) study showed that processing of emotion was associated with increased activation in some regions of limbic system, such as hippocampus and amygdala [24]. Some authors have speculated that whereas limbic (amygdala–hippocampus) regions are particularly involved in the emotional response to exteroceptive sensory stimuli [25]. Moreover, such a result was in line with evidence suggesting that the amygdala is specifically sensitive to fearful emotional processing [26]. Therefore, the increased activities in hippocampus and amygdala may be associated with the emotional disorders in the lung cancer patients.

### Decreased brain glucose uptake in NSCLC patients

In the current study, decreased metabolism regions were found in the superior parietal lobule, inferior parietal lobule, and left fusiform gyrus. In a previous PET/CT study of Nonokuma and colleagues [27], the cerebral glucose metabolism was decreased in the bilateral medial frontal and temporal cortices in lung cancer patients, which may be complement of our study. These hypometabolic regions are major components of the dorsal attention network (DAN), which control the attentional allocation and spatial orientation functions [16]. Reddick reported that survivors of childhood acute lymphoblastic leukemia had significant deficits in attention [28]. D’Agata and colleagues reported that the DAN FDG metabolism was decreased in patients with lymphoma in a PET/CT study [29]. A functional MRI study showed that the functional connectivity in DAN was impaired in women with breast cancer at 1 month after chemotherapy [30]. The above studies indicate that the patients with malignancy experience the deficits in attention, which lead to an abnormal DAN. Therefore, the hypometabolism in DAN and visuospatial function regions might reflect the attention and spatial orientation function changes in patients with lung cancer.

In this study, no significant differences were found in brain glucose uptake among clinical stages or between adenocarcinoma and squamous carcinoma patients. These results were partly in line with the study of Li and colleagues [31]. However, the effect of clinical stages or pathological types on brain glucose uptake might be related to the whole-body tumor burden. The total glycolytic volume (TGV) in lymphoma [32] and lung cancer [27] patients have been demonstrated with a negative correlation to brain glucose uptake. Therefore, the mechanism of interaction between brain metabolism and tumor-related influence on whole body needs further research.

The current study presents several limitations. The psychological and cognitive test of the study subjects was not obtained, and the effects of these factors could not be adequately assessed. Also, the number of patients with small-cell carcinoma was not sufficient and was not brought in for detecting the effect of pathological types on brain glucose uptake. This retrospective study preliminarily explored the glucose metabolic pattern on tumor-to-brain effect. The mechanism of neuromodulation to peripheral tumors needs a prospective and large sample of multi-disciplinary collaborative research in the future.

In conclusion, our current study reveals brain glucose metabolism changes in NSCLC patients. These changes might be attributed to lung-cancer related visceral sympathetic activation and decrease of DAN function. As these deficits are clinically unobvious, the findings in our research have a significant impact for management of patients with lung cancer.

## Supporting Information

S1 Strobe Checklist (DOCX)
